# Pretreatment of Ascorbic Acid Inhibits MPTP-Induced Astrocytic Oxidative Stress through Suppressing NF-*κ*B Signaling

**DOI:** 10.1155/2020/8872296

**Published:** 2020-11-17

**Authors:** Xiaokang Zeng, Kai Xu, Ji Wang, Yunqi Xu, Shaogang Qu

**Affiliations:** ^1^Central Laboratory, Shunde Hospital, Southern Medical University (The First People's Hospital of Shunde Foshan), Foshan, 528300 Guangdong, China; ^2^Department of Neurology, Nanfang Hospital, Southern Medical University/The First School of Clinical Medicine, Southern Medical University, Guangzhou, Guangdong 510515, China; ^3^Central Laboratory and Department of Neurology, Shunde Hospital, Southern Medical University (The First People's Hospital of Shunde Foshan), Foshan, Guangdong, China; ^4^Key Laboratory of Mental Health of the Ministry of Education, Southern Medical University, Guangzhou, China

## Abstract

Astrocytes are a major constituent of the central nervous system (CNS). Astrocytic oxidative stress contributes to the development of Parkinson's disease (PD). Maintaining production of antioxidant and detoxification of reactive oxygen and nitrogen species (ROS/RNS) in astrocytes is critical to prevent PD. Study has illuminated that ascorbic acid (AA) stimulates dopamine synthesis and expression of tyrosine hydroxylase in human neuroblastoma cells. However, the role and regulatory mechanisms of AA on detoxification of astrocytes are still unclear. The purpose of our study is in-depth study of the regulatory mechanism of AA on detoxification of astrocytes. We found that AA pretreatment decreased the expression of ROS and inducible nitric oxide synthase (iNOS) in MPP^+^-treated astrocytes. In contrast, the expression levels of antioxidative substances—including superoxide dismutase (SOD), glutathione (GSH), and glutamate-cysteine ligase modifier (GCLM) subunit—were upregulated after AA pretreatment in MPP^+^-treated astrocytes. However, inhibition of NF-*κ*B prevented such AA induced increases in antioxidative substances following MPP^+^ treatment in astrocytes, suggesting that AA improved antioxidative function of astrocytes through inhibiting NF-*κ*B-mediated oxidative stress. Furthermore, *in vivo* studies revealed that AA preadministration also suppressed NF-*κ*B and upregulated the expression levels of antioxidative substances in the midbrain of MPTP-treated mice. Additionally, pretreatment of AA alleviated MPTP-induced PD-like pathology in mice. Taken together, our results demonstrate that preadministration of AA improves the antioxidative function of astrocytes through suppressing NF-*κ*B signaling, following alleviated the pathogenesis of PD which induced by MPTP. Hence, our findings elucidate a novel protective mechanism of AA in astrocytes.

## 1. Introduction

Parkinson's disease (PD) is the second-most prevalent neurodegenerative disease following Alzheimer's disease and is the most common movement-disorder disease worldwide [1]. Approximately 2% of individuals over 65 years old are afflicted with PD. The prevention situation of PD is very grim. The motor symptoms of PD include bradykinesia, cogwheel rigidity, resting tremor, a slow shuffling gait, and difficulty maintaining balance. Nonmotor symptoms of PD include cognitive decline, depression, anxiety, and sleep disturbances [2]. The pathological hallmarks of PD include depigmentation of the substantia nigra, dopaminergic neuronal loss in the pars compacta of the substantia nigra, and formation of Lewy bodies/cytoplasmic inclusions containing fibrillar *α*-synuclein in the substantia nigra compacta (SNpc) [3]. Currently, levodopa coupled with a DOPA decarboxylase inhibitor is the most effective therapy for PD. However, there are some side effects during levodopa therapy, such as dizziness and gastrointestinal disturbances. Thus, there is a continued need to further identify and develop novel therapeutic agents for PD [4].

Astrocyte is the major class of glial cells in the CNS, providing critical structural and metabolic support for neurons. However, growing evidence shows that oxidative stress of astrocytes contributes to PD pathogenesis. Astrocytes may play a key role in the pathogenesis of PD via oxidative and nitrosative stress [5]. Postmortem analysis of the brains of PD patients and experimental animal models indicate that astrocyte activation and elevated ROS/RNS levels are the pathogenic features of PD [6]. Toxins can trigger excessive formation of reactive oxygen species (ROS), the byproducts of oxygen metabolism including superoxides, hydroxyl radicals, and nitric monoxide, play essential roles in cell signaling, gene transcription, and microbial defense [7]. When the levels of ROS exceed the detoxification capacity of the astrocytic antioxidant system, it leads to pathological oxidative stress [8]. Exposure to environmental toxicants such as 1-methyl-4-phenyl-1,2,3,6-tetrahydropyridine (MPTP), rotenone, and manganese (Mn) may induce reactive astrogliosis and subsequent astrocytic oxidative/nitrosative stress [9–11]. Therefore, elucidating the role of astrocytic oxidative/nitrosative stress in the pathogenesis of PD may greatly expand our understanding of PD. Although the contribution of astrocytic oxidative stress to PD needs to elucidated, the presence of oxidative metabolites in PD patients is related to the in vitro aggregation of *α*-synuclein [12]. In the SNpc of PD patients, the level of the antioxidant molecule GSH secreted mainly by astrocytes is significantly decreased, and the activities of astrocyte-secreted antioxidant enzymes such as SOD, GSH peroxidase (GSH-Px), and catalase (CAT) are significantly decreased compared to healthy controls [13]. Taken together, these findings suggest that there is a need to continue to explore treatments for PD by targeting oxidative/nitrosative stress in astrocytes.

Ascorbic acid (AA) has many different functions in the CNS, including the following: (1) it crosses the blood-brain barrier and has been tested as a potential drug for treating degenerative diseases; (2) it promotes neuronal differentiation, maturation, and survival; and (3) it participates in catecholaminergic synthesis and modulates neurotransmission [14]. However, its role in the astrocytic oxidative stress is still clear. Lee et al. found that AA promoted differentiation of astrocytes by enhancing the expression of NeuroD, Notch, bone morphogenetic protein 2 (BMP2), and bone morphogenetic protein 7 (BMP7) [15]. Secretion of brain-derived neurotrophic factor (BDNF) by astrocytes is also increased after administration of AA *in vitro*, which contributes to cellular survival by enhancing the expression of SOD and GSH [16]. However, there are some different views, Kao et al. found that rat brain astrocytes (RBA-1 cells) incubated with AA for 2 days or 7 days, the SOD activity, SOD mRNA level, and SOD protein level were significantly decreased [17]. But Sanchez-Moreno et al. found that AA has a protective effect against the ethanol-mediated toxic effects on human brain glial cells by reducing the expression of cyclooxygenase-2 (COX-2) and synthesis of prostaglandin E2 (PGE2) [18]. Thus, we hypothesize that AA may have a protective effect on PD by inhibiting astrocytic oxidative stress.

In the present study, we revealed that AA decreased ROS production in astrocytes *in vitro* via inhibition of NF-*κ*B-mediated oxidative stress. Pretreatment of AA improved behavioral performance and restored tyrosine dehydrogenase expression in MPTP-treated mice *in vivo*. Thus, our findings demonstrated that pretreatment of AA alleviated MPTP-induced PD symptoms through inhibiting astrocytic oxidative stress. Collectively, our findings unveil a protective role of AA in astrocytic oxidative stress and suggest that AA may represent a therapeutic drug for PD.

## 2. Materials and Methods

### 2.1. Animals and Treatments

The protocols used for animal experiments were approved by the Institutional Animal Care and Use Committee of Southern Medical University. All procedures involving animals were approved by the Animal Ethics Committee of Southern Medical University. Male C57BL/6 mice (8-week old) were used in our experiments. The mice were from Guangdong Medical Animal Laboratory (Foshan, China). All animals were provided food and water *ad libitum*, under a 12/12-hour light/dark cycle. Age- and weight-matched animals were randomly assigned to each group. Animal experiments were conducted in the following four groups (*n* = 20 for each group): a saline-treated control group, AA-treated group, MPTP-treated group, and AA+MPTP-treated group. AA (Sigma, A7506, MO, US) was dissolved in saline solution. Mice received intraperitoneal injections of AA at 100 mg/kg every three days for 60 days. After supplementation with AA for two months, mice simultaneously received AA and intraperitoneal injections of MPTP (Sigma, M0896, MO, US) every three days (25 mg/kg in saline) for 35 days, the animals received AA during MPTP injection. After MPTP lesion, behavioral experiments were conducted. Subsequently, mice were euthanized, and their midbrains were quickly dissected for analyses in various assays. The mortality rate in the MPTP-treated group was approximately 20%; however, no mice died in any of the other groups.

### 2.2. Cell Culture

Primary midbrain astrocytes were obtained from one-day-old C57BL/6 pups, as previously described by our group [19]. Mice pups were anesthetized with isoflurane and then the cerebral hemispheres were removed, after which midbrains were collected in ice-cold phosphate-buffered solution (PBS). Tissue was thoroughly chopped with scissors and was then incubated in 0.25% trypsin–EDTA for 10 min at 37°C. DMEM/F12 (Gibco, 11330057, MA, US) supplemented with 10% fetal bovine serum (FBS) (Gibco, 26010074, MA, US) was then added to stop digestion. After centrifugation (1,000 rpm) for 5 min, each pellet was resuspended and seeded into a T75 flask (Corning, 43072, NY, US). The culture medium was changed every three days. Cells were cultured for seven days, and the microglia and oligodendrocytes were then removed by thermostatic oscillation at 200 rpm for 18 h. Astrocytes were identified by immunofluorescence with an antibody against glial fibrillary acidic protein (GFAP) (CST, #3670, MA, US).

### 2.3. Detection of Intracellular Reactive Oxygen Species

Intracellular ROS were detected by a 2,7dichlorodi-hydrofluorescein diacetate (DCFH-DA) fluorescence assay (Beyotime, S003M, Shanghai, China). Briefly, astrocytes were seeded into confocal dish at a density of 2 × 10^5^ cells/well and were cultured overnight. After treatment with AA and/or MPTP, cells were incubated in serum-free medium containing 10 mM of DCFH-DA at 37°C in an incubator for 6 h. Each dish was subsequently washed three times with cold PBS to remove unbound DCFH-DA. Images were acquired vial laser-scanning confocal microscopy (Leica, SP8, Wetzlar, Germany), and the same settings were used for all samples in each experiment. Fluorescent intensities were calculated using the Image-Pro Plus 6.0 software (Silver Spring, MD, US).

### 2.4. Immunohistochemistry

For immunohistochemistry, mice were anesthetized and transcardially perfused with both freshly prepared PBS and, subsequently, 4% paraformaldehyde (Sangon, E672002, Shanghai, China) in PBS. After mice were decapitated, their brains were dissected. Brain tissue samples were embedded in optimum-cutting temperature compound (Leica, DJ-LDB-004, Nussloch, Germany) and stored at -80°C. Serial 10 *μ*m-thick coronal tissue sections were cut using a freezing microtome (Leica, CM1950, Nussloch, Germany). Citrate buffer (Sangon, E673002, Shanghai, China) was added to the sections to retrieve antigens. Sections were treated with 3% hydrogen peroxide (Sigma, 323381, MO, USA) for 10 min to enable penetration and were then blocked with 5% BSA (Sangon, E661003, Shanghai, China) for 30 min at room temperature. Sections were incubated overnight at 4°C with primary antibodies as follows: tyrosine hydroxylase (TH) antibody (Santa Cruz, sc-25269). After washing three times with PBS (5 min each wash). Sections were incubated sequentially in HRP-conjugated goat anti-mouse (Beyotime, A0208) and goat anti-rabbit secondary antibody (Beyotime, A0216) for 2 h at 37°C. Sections were visualized with a 3,3-diaminobenzidine (DAB) peroxidase substrate kit (Boster, AR1022). Integrated optical density (IOD) was determined using an Image-Pro Plus 6.0 photogram analysis system (IPP 6.0; Media Cybernetics, Bethesda, MD, US).

### 2.5. Western Blotting

Cells or midbrain tissues were added to 100 *μ*l/10^6^ cells or 500 *μ*l/g tissue of RIPA buffer (Beyotime, P0013E) containing 1 mM of phenylmethanesulfonyl fluoride (PMSF) (Beyotime, ST506) and were then lysed for 1 h on ice. Extracts were centrifuged for 10 min at 14,000 rpm. Protein concentrations were measured by BCA assays (Beyotime, P0012S). Samples were diluted with protein loading buffer and heated to 95°C for 10 min and were stored at -80°C prior to Western blotting. Protein extracts were separated by SDS-PAGE on 12% polyacrylamide gels and were subsequently electrophoretically transferred to a PVDF membrane (Millipore, GVHP29325). After blocking with 5% (w/v) BSA in Tris-buffered saline with tween (TBST) at room temperature for 2 h, membranes were then incubated with an appropriate specific primary antibody from Santa Cruz (anti-TH, sc-25269; anti-SOD1, sc-101523; anti-gp91phox, sc-130543; anti-p47phox, sc-17844; anti-GCLM, sc-55586; anti-HO-1, sc-390991; anti-PGC-1*α*, sc-518025; anti-pp65, sc-166748; anti-p65, sc-8008; anti-pi*κ*B*α*, sc-52943; anti-i*κ*B*α*, sc-1643; anti-pp38, sc-166182; anti-p38, sc-81621; anti-p-ERK1/2, sc-81492; anti-ERK1/2, sc-135900; anti-pJNK1/2, sc-293136; anti-JNK1/2, sc-137019; and anti-*β*-actin, sc-8432) at 4°C overnight, followed by incubation with an HRP-conjugated secondary antibody [anti-mouse, -rabbit, or -goat (Beyotime, A0208; A0216); or anti-sheep, (Abcam, ab6764)]. Detection was performed using an enhanced chemiluminescence kit (Thermo Scientific, 32106) according to the manufacturer's instructions. For analysis of phosphorylated protein, total protein was set as a loading control, and for other protein, *β*-actin was set as a loading control. Protein-band density values of total proteins were normalized to *β*-actin, and protein-band density values of phosphorylated proteins were normalized to their total proteins. Protein-band densities were measured using the ImageJ software. Data were collected from at least three independent experiments.

### 2.6. Determination of Intracellular Superoxide Dismutase and Glutathioneactivity

Total SOD and GSH activities in cell culture supernatants were determined using total SOD and GSH assay kits with WST-8 (Beyotime, S0101M; S0053), based on the protocols provided by the manufacturer [20].

### 2.7. Open-Field Test

Locomotion behavior was evaluated in a square open field [21]. The open-field apparatus consisted of a black PVC box (Ugo Basile, Italy) with the following dimensions: 44cm(l) × 44cm(w) × 30cm(h). The floor was divided into nine equal squares using brightly colored tape to facilitate quantification of locomotion. Mice were transported to the testing room before testing. For each test, pups were placed in the center of the box and allowed to explore the arena for 1 min.

### 2.8. Rotarod Test

Motor coordination and balance were assessed by an automated four-lane rotarod unit (Ugo Basile, Italy) every three days after MPTP lesion. The mice were pretrained for three days (at 8 rpm on day 1, at 10 rpm on day 2, and 12 rpm on day 3) to reach a stable performance. The mice were placed on the rod, and the rotation speed was started at 4 rpm and was then accelerated to 40 rpm within 2 min. The latency to fall from the rod was automatically recorded.

### 2.9. Grip Strength Test

Muscle strength in all four limbs was determined using a grip strength meter (Ugo Basile, 47200, Italy) after MPTP-induced lesions.

### 2.10. Pole Test

Mice were placed on the peak of a foam ball (diameter: 2.0 cm) fixed on a stick (diameter: 1.0 cm; length: 50 cm). The climbing time from the peak to the bottom of the stick was then recorded for each mouse, and each mouse had to have at least three successful trials.

### 2.11. Statistical Analysis

All experimental results are expressed as the mean ± standarddeviation (SD) and were plotted using the GraphPad Prism 7 software (IBM Corp., Armonk, NY, US). Statistical analysis of data was performed by LSD or Dunnett's posttests based on the ANOVA for multivariate data analysis using SPSS 22.0 (SPSS Inc., Chicago, IL, US). A *P* value of less than 0.05 was considered statistically significant (^∗^ denotes significance compared to control: ^∗^*P* < 0.05, ^∗∗^*P* < 0.01, and ^∗∗∗^*P* < 0.001; ^#^ denotes significance compared to MPTP or MPP^+^: ^#^*P* < 0.05, ^##^*P* < 0.01, and ^###^*P* < 0.001).

## 3. Results

### 3.1. Ascorbic Acid Reduces MPP^+^-Induced Reactive Oxygen Species and Inducible Nitric Oxide Synthase Production and Enhances Antioxidant Expression in Astrocytes In Vitro

**Figure 1:**
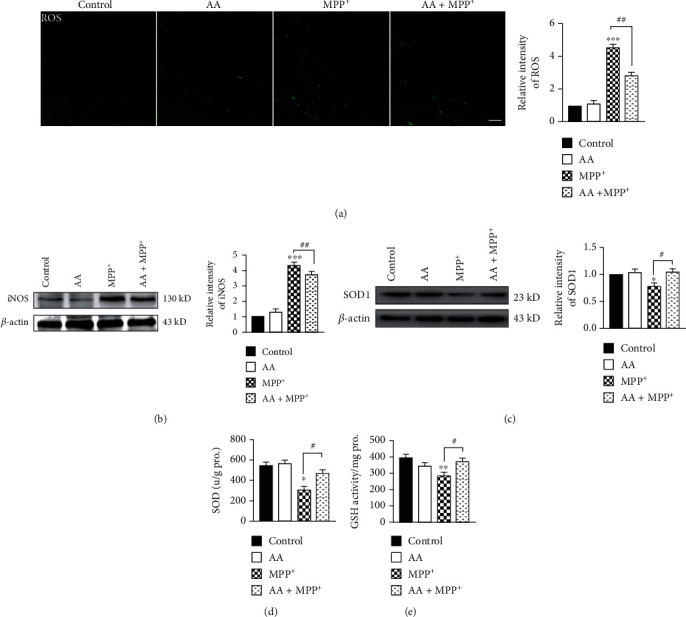
Ascorbic acid reduces MPP^+^-induced reactive oxygen species (ROS) and inducible nitric oxide synthase (iNOS) production and increases antioxidant expression in astrocytes *in vitro*. Primary astrocytes were treated with 1 mM of AA for 24 h and/or 1 mM of MPP^+^ for another 24 h. (a) Intracellular ROS was measured using the carboxy-H2DCFDA method (scale bar, 20 *μ*m) (*n* = 3). (b) The expression of iNOS was detected by Western blotting (*n* = 3). (c) The expression of SOD1 was detected by Western blotting (*n* = 3). (d) The concentration of SOD in the supernatant from primary astrocytes was detected by ELISAs (*n* = 3). (e) The concentration of GSH in the supernatant from primary astrocytes was detected by ELISAs (*n* = 3). Data were obtained from three independent experiments. One-way ANOVAs followed by LSD pairwise comparisons were performed. ^∗^ was considered significant compared to control (^∗^*P* < 0.05, ^∗∗^*P* < 0.01, and ^∗∗∗^*P* < 0.001). ^#^ was considered significant compared to MPTP or MPP^+^ (^#^*P* < 0.05, ^##^*P* < 0.01, and ^###^*P* < 0.001).

MPP^+^ is known to induce oxidative stress *in vitro*. After MPP^+^ treatment, a large quantity of ROS accumulated in primary astrocytes *in vitro*. To examine whether AA prevents the production of ROS after MPP^+^ treatment, the accumulation of ROS in astrocytes was measured using the fluorescent probe, DCFH-DA. We found that 1 mM of MPP^+^ induced increased intracellular ROS production in astrocytes after 24 h of treatment. However, pretreatment with AA inhibited MPP^+^-induced production of ROS (Figure 1(a)). Oxidative stress plays an important role in the onset of PD, as it induces glial cells to overexpress inducible nitric oxide synthase (iNOS), which results in the production of toxic levels of NO in neurons. In our present study, we found that after MPP^+^ treatment, the iNOS expression was significantly increased. However, AA pretreatment significantly decreased MPP^+^-induced iNOS expression compared to that of the MPP^+^ group not receiving AA (Figure 1(b)). The antioxidant system is responsible for removing oxidizing substances. The main antioxidant enzymes are SOD, POD, and glutathione reductase. We found that the expression of SOD1 was decreased in astrocytes after MPP^+^ treatment. In contrast, AA pretreatment significantly increased SOD1 expression compared to that of the MPP^+^ group not receiving AA (Figure 1(c)). We also detected antioxidants in the supernatant of astrocytes. We found that the released levels of SOD and GSH from astrocytes in the AA-pretreated MPP^+^ group were higher than those in the MPP^+^ group (Figures 1(d) and 1(e)). These results indicate that AA improved antioxidative function in MPP^+^-treated astrocytes.

### 3.2. Ascorbic Acid Inhibits MPP^+^-Induced Nicotinamide Adenine Dinucleotide Phosphate (NADPH) Activation and Enhances the Expression of Antioxidative Proteins in Astrocytes In Vitro

**Figure 2:**
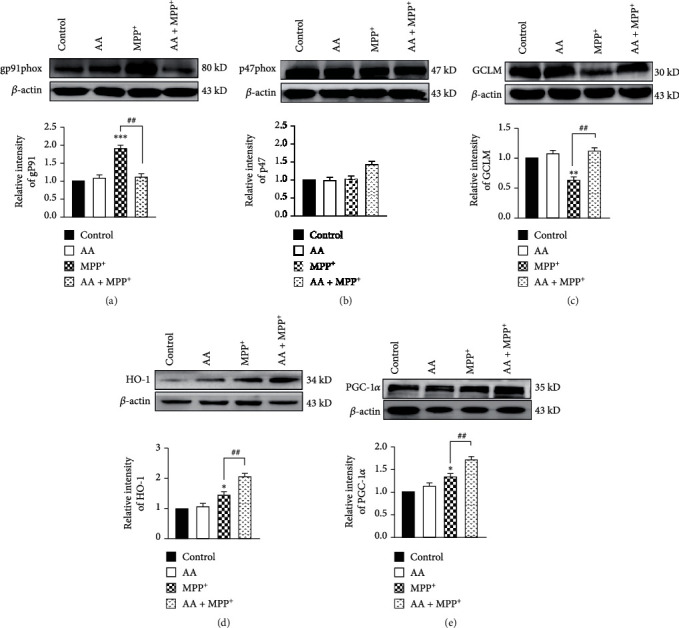
Ascorbic acid inhibits MPP^+^-induced nicotinamide adenine dinucleotide phosphate (NADPH) oxidase activation and enhances the expression of antioxidative proteins in astrocytes *in vitro*. Primary astrocytes were treated with 1 mM of AA for 24 h and/or 1 mM of MPP^+^ for another 24 h. (a) The expression of total gp91phox was detected by Western blotting (*n* = 3). (b) The expression of total p47phox was detected by Western blotting (*n* = 3). (c) The expression of GCLM was detected by Western blotting (*n* = 3). (d) The expression of HO-1 was detected by Western blotting (*n* = 3). (e) The expression of PGC-1*α* was detected by Western blotting (*n* = 3). Data were obtained from three independent experiments. One-way ANOVAs followed by LSD pairwise comparisons were performed. ^∗^ was considered significant compared to control (^∗^*P* < 0.05, ^∗∗^*P* < 0.01, and ^∗∗∗^*P* < 0.001). ^#^ was considered significant compared to MPTP or MPP^+^ (^#^*P* < 0.05, ^##^*P* < 0.01, and ^###^*P* < 0.001).

Many glial enzymes are involved in the formation of ROS. NADPH oxidase plays an important role in producing ROS in the brain. In our present study, we found that the NADPH oxidase subunit, gp91phox, was activated after MPP^+^ treatment. In contrast, AA pretreatment reduced the MPP^+^-induced expression of gp91phox. However, the expression of another NADPH oxidase subunit, p47phox, was not changed from any treatment (Figures 2(a) and 2(b)). These results suggest that AA decreased MPP^+^-induced NADPH oxidase activity through inhibiting gp91phox expression. Glutamate-cysteine ligase modifier (GCLM) subunit belongs to the aldo/keto reductase family. GCLM catalyzes the first step of GSH synthesis. In our present study, we found that MPP^+^ treatment decreased the expression of GCLM in astrocytes after 24 h of stimulation, which was reversed by AA pretreatment (Figure 2(c)). Heme oxygenase-1 (HO-1) is expressed in many cell types, including astrocytes, and has been identified as an important endogenous protective factor induced by various stimulants, such as oxidative stress, heat shock, and endotoxins. Herein, we found that the expression of HO-1 in astrocytes exposed to MPP^+^ was significantly increased. Compared to that in the MPP^+^ group, the expression of HO-1 was significantly increased in the AA-pretreated MPP^+^ group (Figure 2(d)). Peroxisome proliferator-activated receptor gamma coactivator-1 alpha (PGC-1*α*) is a newly characterized transcriptional regulator that plays a key role in antioxidant stress systems. To investigate the effect of AA on PGC-1*α*, we examined the protein level of PGC-1*α* in astrocytes. Treatment with 0.5 mM of MPP^+^ increased the expression of PGC-1*α*. Preadministration of AA could further enhanced MPP^+^-induced expression of PGC-1*α* (Figure 2(e)). These results indicate that AA inhibited NADPH oxidase and upregulated the expression of antioxidative proteins in MPP^+^-treated astrocytes *in vitro*.

### 3.3. Ascorbic Acid Inhibits MPP^+^-Induced Nuclear Factor-*κ*B (NF-*κ*B) Activation but Does Not Affect Mitogen-Activated Protein Kinasep38/Extracellular-Regulated Protein Kinases (ERK) or c-Jun N-Terminal Kinase (JNK) Signaling in Astrocytes In Vitro

**Figure 3:**
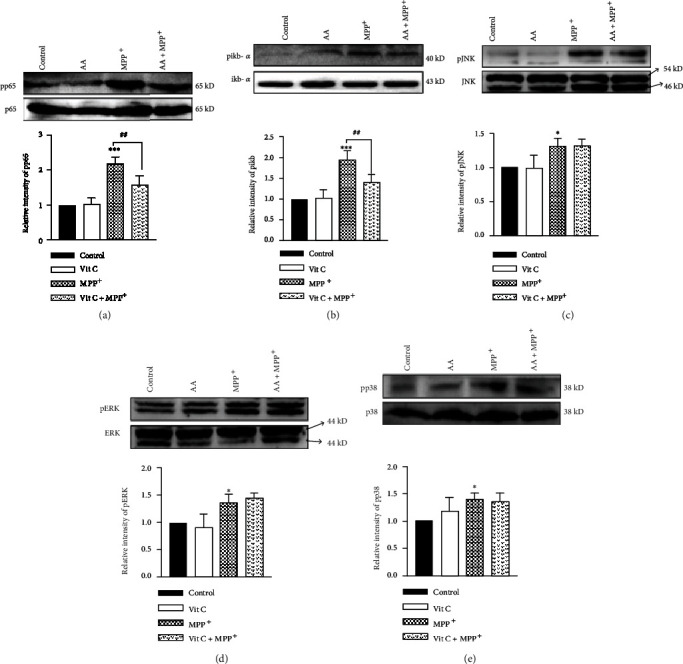
Ascorbic acid inhibits MPP^+^-induced nuclear factor-kappaB (NF-*κ*B) activation but does not affect p38/ERK or JNK signaling in astrocytes *in vitro*. Primary cells were treated with 1 mM of AA for 24 h and/or 1 mM of MPP^+^ for another 24 h. (a) The expression of phosphorylated p65 (pp65) was detected by Western blotting (*n* = 3). (b) The expression of phosphorylated i*κ*B*α* (pi*κ*B*α*) was detected by Western blotting (*n* = 3). (c) The expression of phosphorylated JNK (pJNK) was detected by Western blotting (*n* = 3). (d) The expression of phosphorylated ERK (pERK) was detected by Western blotting (*n* = 3). (e) The expression of phosphorylated p38 (pp38) was detected by Western blotting (*n* = 3). Data were obtained from three independent experiments. One-way ANOVAs followed by LSD pairwise comparisons were performed. ^∗^ was considered significant compared to control (^∗^*P* < 0.05, ^∗∗^*P* < 0.01, and ^∗∗∗^*P* < 0.001). ^#^ was considered significant compared to MPTP or MPP^+^ (^#^*P* < 0.05, ^##^*P* < 0.01, and ^###^*P* < 0.001).

In our present study, we found that AA decreased the MPP^+^-induced activation of NADPH oxidase, which inhibited the production of ROS and eventually led to increased expression of antioxidant proteins. However, which signal pathways are involved in this process remain unclear. Therefore, we next assayed the classical MAPK signaling pathway and its downstream NK-*κ*B signaling pathway. We found that MPP^+^ stimulation elevated the expression levels of phosphorylated p65 and i*κ*B*α*. However, preadministration with AA reversed the MPP^+^-induced expression of phosphorylated p65 and i*κ*B*α* (Figures 3(a) and 3(b)). MPP^+^ treatment elevated the expression levels of phosphorylated JNK, ERK, and p38, but pretreated with AA did not change any of these MPP^+^-induced changes in expression levels (Figures 3(c)–3(e)). These results indicate that AA administration decreased the MPP^+^-induced activation of p65, decreasing the level of astrocytic oxidative stress.

### 3.4. Inhibition of NF-*κ*B Signaling Blocks the Antioxidative Effects of Ascorbic Acid and Perpetuates Cytotoxicity in MPP^+^-Treated Astrocytes In Vitro

**Figure 4:**
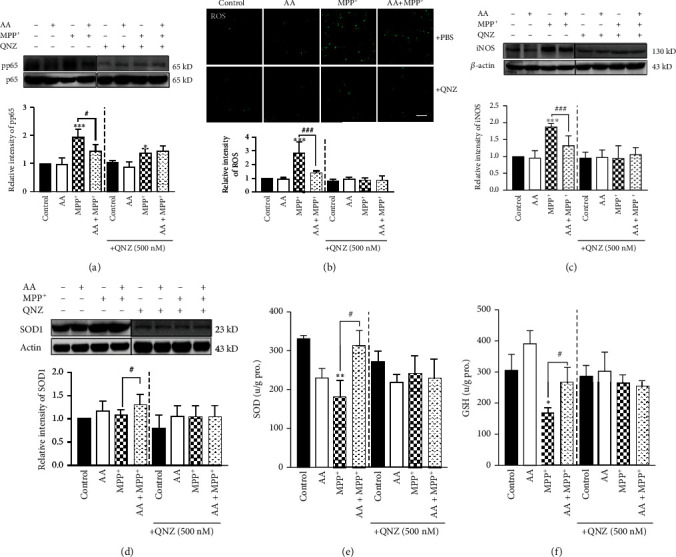
Inhibition of NF-*κ*B signaling blocks the antioxidative effects of ascorbic acid and perpetuates cytotoxicity in MPP^+^-treated astrocytes *in vitro*. Primary astrocytes were copretreated with 1 mM of AA and 0.5 mM of QNZ (EVP4593) for 24 h and/or 1 mM of MPP^+^ for another 24 h. (a) The NF-*κ*B-inhibition efficiency was detected by measuring the phosphorylation of p65 by Western blotting (*n* = 3). (b) Intracellular ROS was measured using the carboxy-H2DCFDA method (scale bar, 100 *μ*m) (*n* = 3). (c) The expression of iNOS was detected by Western blotting (*n* = 3). (d) The expression of SOD1 was detected by Western blotting (*n* = 3). (e) The concentration of SOD in the supernatant from primary astrocytes was detected by ELISAs (*n* = 3). (f) The concentration of GSH in the supernatant from primary astrocytes was detected by ELISAs (*n* = 3). Data were obtained from three independent experiments. One-way ANOVAs followed by LSD pairwise comparisons were performed. ^∗^ was considered significant compared to control (^∗^*P* < 0.05, ^∗∗^*P* < 0.01, and ^∗∗∗^*P* < 0.001). ^#^ was considered significant compared to MPTP or MPP^+^ (^#^*P* < 0.05, ^##^*P* < 0.01, and ^###^*P* < 0.001).

To determine the role of NF-*κ*B in the antioxidation of AA in MPP^+^-treated astrocytes, we administrated QNZ (Selleck, EVP4593) to inhibit NK-*κ*B activation. We found that after QNZ administration, MPP^+^-induced expression of phosphorylated p65 was decreased significantly (Figure 4(a)). Next, we tested whether inhibition of NF-*κ*B affected the production of ROS in astrocytes. We found that after QNZ administration, the production of ROS in astrocytes was significantly reduced in the MPP^+^ group but AA-pretreatment MPP^+^ group was unaffected in the control group and AA group (Figure 4(b)). We also detected the expression of iNOS in astrocytes with or without NF-*κ*B inhibition. The results showed that the expression of iNOS was decreased in the AA-pretreatment MPP^+^ group compared to that in the MPP^+^ group. However, the expression of iNOS was comparable between the AA-pretreatment MPP^+^ group and MPP^+^ group after NF-*κ*B inhibition (Figure 4(c)). Since the MPP^+^-induced productions of ROS and iNOS were reduced after NF-*κ*B inhibition, we next investigated whether inhibition NF-*κ*B disturbs the expression of antioxidative enzymes in MPP^+^-treated astrocytes. We found that the MPP^+^-induced expression of SOD1 was reduced after NF-*κ*B inhibition. Additionally, there was no significant difference in SOD1 expression between the AA-pretreatment MPP^+^ group and MPP^+^ group following NF-*κ*B inhibition (Figure 4(d)). Furthermore, we determined whether NF-*κ*B inhibition changed the release of SOD and GSH from astrocytes. We found that the MPP^+^-induced levels of released SOD1 and GSH were decreased after NF-*κ*B inhibition. Furthermore, there was no difference in SOD and GSH levels between the AA-pretreatment MPP^+^ group and MPP^+^ group following NF-*κ*B inhibition (Figures 4(e) and 4(f)). These results indicate that AA exerted antioxidative effects in MPP^+^-treated astrocytes *in vitro* through NF-*κ*B signaling.

### 3.5. Inhibition of NF-*κ*B Signaling Blocks the Protective Effects of Ascorbic Acid in MPP^+^-Induced Astrocytes In Vitro through Suppressing the Expression of Antioxidative Proteins

**Figure 5:**
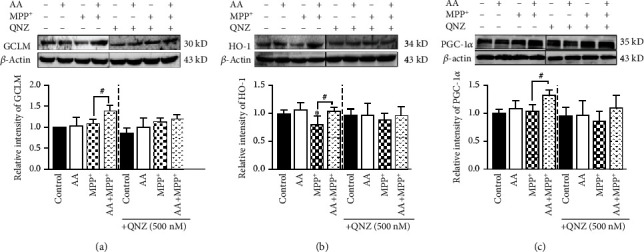
Inhibition of NF-*κ*B signaling suppresses the expression of antioxidative proteins and blocks the protective effects of ascorbic acid to perpetuate cytotoxicity in MPP^+^-treated in astrocytes *in vitro*. Primary astrocytes were copretreated with 1 mM of AA and 0.5 mM of QNZ (EVP4593) for 24 h and/or 1 mM of MPP^+^ for another 24 h. (a) The expression of GCLM was detected by Western blotting (*n* = 3). (b) The expression of HO-1 was detected by Western blotting (*n* = 3). (c) The expression of PGC-1*α* was detected by Western blotting (*n* = 3). Data were obtained from three independent experiments. One-way ANOVAs followed by LSD pairwise comparisons were performed. ^∗^ was considered significant compared to control (^∗^*P* < 0.05, ^∗∗^*P* < 0.01, and ^∗∗∗^*P* < 0.001). ^#^ was considered significant compared to MPTP or MPP^+^ (^#^*P* < 0.05, ^##^*P* < 0.01, and ^###^*P* < 0.001).

We demonstrated that AA exerted its role in inhibiting MPP^+^-induced oxidative stress via the NF-*κ*B pathway. Next, we assessed whether blocking NF-*κ*B disrupted the expression of antioxidative proteins. We first inhibited p65 phosphorylation by QNZ, and then administered AA and/or MPP^+^ to primary astrocytes *in vitro*. Finally, we collected the total protein from each group and detected the expression of GCLM, HO-1, and PGC-1*α* via Western blotting. We found that inhibition of NF-*κ*B did not affect the expression of these proteins under control conditions. However, inhibition of NF-*κ*B eliminated the differences in expression levels of GCLM, HO-1, and PGC-1*α* between the AA-pretreatment MPP^+^ group and MPP^+^ group (Figures 5(a)–5(c)). These results suggest that blocking NF-*κ*B signaling occluded the protective effects of AA in MPP^+^-treated astrocytes through suppressing the expression of antioxidative proteins.

### 3.6. Ascorbic Acid Enhances Antioxidant Function through Decreasing NK-*κ*B Activation in the Midbrain of MPTP-Treated Mice

**Figure 6:**
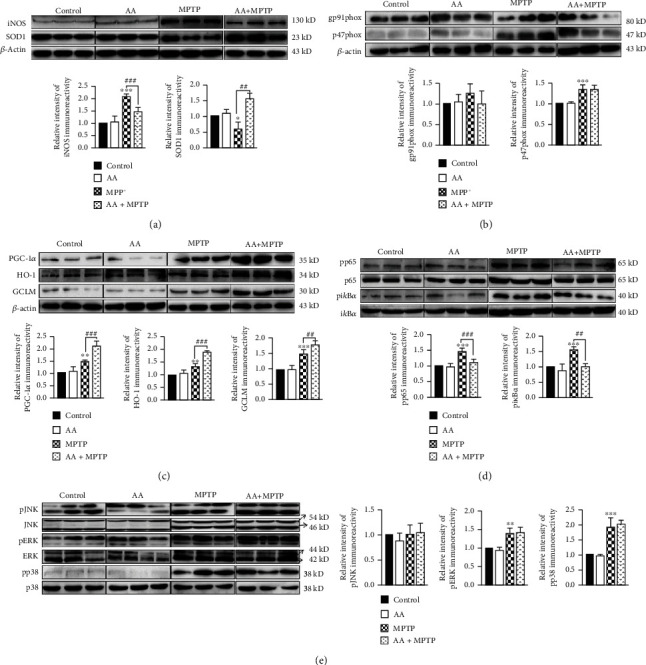
Ascorbic acid enhances antioxidant function and decreases NK-*κ*B activation in the midbrains of MPTP-treated mice. Wild-type mice were intraperitoneally treated with 500 mg/kg of AA for 60 days, following by coinjected with 25 mg/kg of MPTP every 3.5 days for five weeks. After behavioral testing, midbrains were removed and total proteins were extracted. (a) The expression levels of iNOS and SOD1 were detected by Western blotting (*n* = 3). (b) The expression of gp9phox and p47phox was detected by Western blotting (*n* = 3). (c) The expression levels of PGC-1*α*, HO-1, and GCLM were detected by Western blotting (*n* = 3). (d) The expression levels of phosphorylated p65 and i*κ*B*α* were detected by Western blotting (*n* = 3). (e) The expression levels of phosphorylated JNK, ERK, and p38 were detected by Western blotting (*n* = 3). Data were obtained from three independent experiments. One-way ANOVAs followed by LSD pairwise comparisons were performed. ^∗^ was considered significant compared to control (^∗^*P* < 0.05, ^∗∗^*P* < 0.01, and ^∗∗∗^*P* < 0.001). ^#^ was considered significant compared to MPTP or MPP^+^ (^#^*P* < 0.05, ^##^*P* < 0.01, and ^###^*P* < 0.001).

Our *in vitro* experiments demonstrated that AA pretreatment improved antioxidant function and reduced oxidative stress in MPP^+^-treated astrocytes via increasing expression levels of GCLM, HO-1, and PGC-1*α* via inhibition of the NK-*κ*B signal pathway. However, the molecular mechanisms for such AA-induced neuroprotection *in vivo* remained unclear. Hence, we next assayed relevant signaling molecules in the midbrains of mice from each experimental group. We found that the expression of iNOS was significantly elevated in the MPTP group, whereas this MPTP-induced increase was significantly reduced in the AA-pretreatment MPTP group (Figure 6(a)). On the contrary, the expression the antioxidant protein, SOD1, in the AA-pretreatment MPTP group was significantly higher compared to that in the MPTP group (Figure 6(b)). We further detected the expression levels of several antioxidant proteins—namely, GCLM, HO-1, and PGC-1*α*—via Western blotting and found that MPTP treatment increased these expression levels, while AA pretreatment further increased the MPTP-induced expression levels of GCLM, HO-1, and PGC-1*α* (Figure 6(c)). These results indicated that AA pretreatment enhanced the expression levels of antioxidant proteins and reduced oxidative stress in MPTP-treated mice. We further explored the molecular mechanisms underlying this AA-induced phenotype in MPTP-treated mice. Interestingly, we found that AA pretreatment also inhibited MPTP-induced NF-*κ*B activation in the midbrain (Figure 6(d)). However, AA did not alter MPTP-induced changes in the expression levels of components of the JNK, ERK, and p38 signaling pathways (Figure 6(e)). Collectively, these results indicate that AA inhibits MPTP-induced activation of NF-*κ*B and enhances antioxidant function to protect MPTP-treated mice.

### 3.7. Ascorbic Acid Ameliorates MPTP-Induced Behavioral Deficits and Pathophysiology in Mice

**Figure 7:**
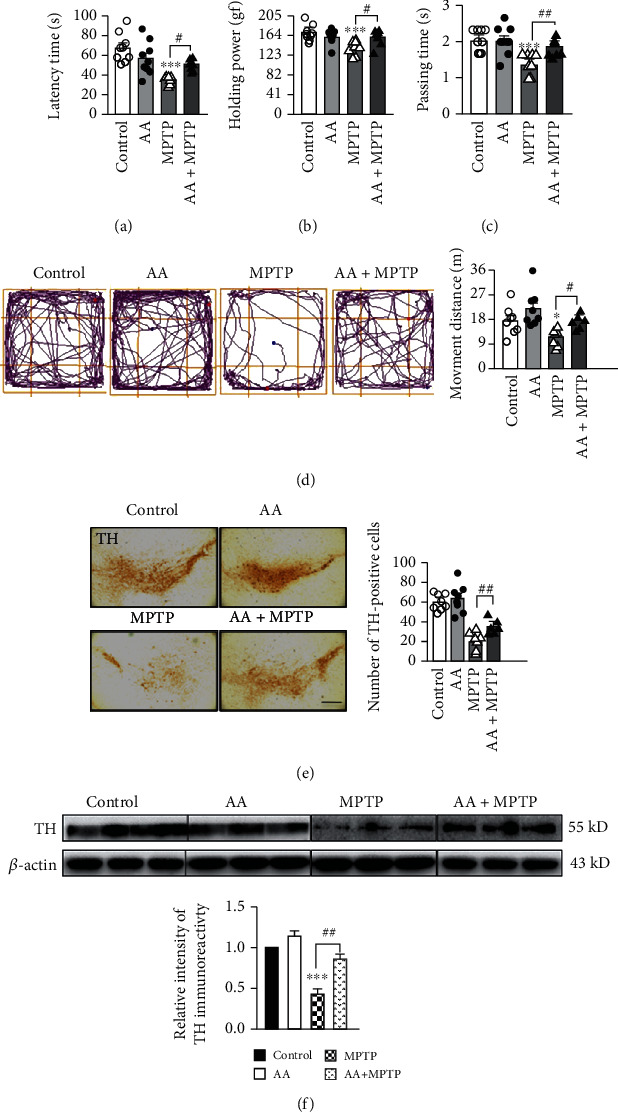
Ascorbic acid pretreatment ameliorates PD-like behavioral deficits and pathophysiology in an MPTP-induced mouse model of PD. (a) Holding times in the rotarod test were recorded after training mice for three days (*n* = 6). (b) Grasping strengths were measured using a grip meter after training mice for three days (*n* = 6). (c) Pole-climbing times were recorded after training mice for three days (*n* = 6). (d) Motion tracking was monitor in the open-field test (*n* = 6). (e) The expression of TH in the midbrain was detected by immunohistochemistry (scale bar, 50 *μ*m) (*n* = 3). (f) The expression level of TH in the midbrain was detected by Western blotting (*n* = 3). Data were obtained from three independent experiments. One-way ANOVAs followed by LSD pairwise comparisons were performed. ^∗^ was considered significant compared to control (^∗^*P* < 0.05, ^∗∗^*P* < 0.01, and ^∗∗∗^*P* < 0.001). ^#^ was considered significant compared to MPTP or MPP^+^ (^#^*P* < 0.05, ^##^*P* < 0.01, and ^###^*P* < 0.001).

To explore the effects of AA on behavioral performance in MPTP-treated mice, we tested mice in the rotarod test, grip power test, pole test, and open-field test. Mice were assigned to the following four treatment groups: saline+vehicle (control), AA+vehicle (AA), MPTP+vehicle (MPTP), and AA+MPTP (AA+MPTP). AA treatment alone had no effects compared to those of control treatment. In contrast, MPTP treatment induced poor behavioral performance, including a reduced holding time in the rotarod test, decreased grip strength in the grip strength test, prolonged pole-climbing time in the pole test, and shortened the movement distance in the open-field test. AA pretreatment in MPTP-treated mice ameliorated MPTP-induced behavioral deficits compared with performances in vehicle-treated MPTP mice (Figures 7(a)–7(d)). We also explored whether AA ameliorated MPTP-induced pathology. We detected tyrosine hydroxylase- (TH-) positive cells in the SNpc via immunohistochemistry. We found that AA pretreatment in MPTP-treated mice significantly reversed MPTP-induced loss of TH-positive cells in the SNpc compared to that of vehicle treatment in MPTP-treated mice (Figure 7(e)). We also confirmed this result by measured total TH expression in the midbrain. The expression of TH in the vehicle-treated MPTP group was significantly lower than that in the control group. In contrast, AA pretreatment restored expression of TH in MPTP-treated mice (Figure 7(f)). These results confirm the protective effects of AA in MPTP-treated mice.

## 4. Discussion

The current understanding of the pathogenesis of PD involves the formation of ROS and the onset of oxidative stress leading to oxidative damage in the SNpc [22]. Extensive postmortem studies have provided evidence to support the involvement of oxidative stress in the pathogenesis of PD; in particular, these forms of oxidative stress include alterations in brain iron content, impaired mitochondrial function, alterations in antioxidant protective systems, and oxidative damage to lipids, proteins, and DNA [23, 24]. Therefore, use of antioxidants is considered to represent a promising approach for inhibiting astrocytic oxidative stress and slowing the progression of PD.

AA is an important antioxidant in the human brain. A previous study reported that frequent AA supplementation can reduce 20% of oxidative damage in the body [14]. During central nervous system (CNS) injury or disease, the overproduction of ROS and/or insufficient detoxification can result in the activation of a coordinated astrocytic response, which involves a series of biochemical and morphological changes collectively referred to as reactive astrogliosis [25]. Reactive astrocytes respond to acute cellular stress and work to limit CNS damage, but chronic astrogliosis can result in the sustained production of ROS and the release of proinflammatory molecules, which promote neuronal injury and neurotoxicity. Our present study found that AA pretreatment reduced MPP^+^-induced ROS production and iNOS expression in astrocytes *in vitro*. Further results indicated that AA pretreatment increased the expression of the antioxidant molecules, SOD and GSH, in MPP^+^-treated astrocytes. These findings indicate that AA reduced MPP^+^-induced excessive ROS levels by increasing antioxidant activities in astrocytes *in vitro*.

A significant source of ROS in PD-like pathology derives from NADPH oxidase, which is a multimeric enzyme composed of gp91phox, p47phox, p22phox, p67phox, and p40phox subunits. Upon cellular activation, p47phox assembles with gp91phox and p22phox, thus forming a NADPH-oxidase entity capable of reducing oxygen to superoxide radicals [26]. A previous study found that the expression of gp91phox was increased in the midbrain of MPTP-treated mice [27]. Additionally, inhibition of gp91phox may prevent MPTP-associated ROS production. A previous study found that NADPH oxidase plays an important role in ROS production [28]. Another study found that NADPH oxidase (NOX2) was upregulated in a rotenone-induced cellular model of PD. Furthermore, inhibition of NOX2-dependent oxidative stress attenuates aberrant autophagy and cellular death in a rotenone-induced cellular model of PD [29]. Lv et al. found that GCLM deletion led to a decrease in GSH in the striatum, indicating that GCLM is important for maintaining GSH expression in the midbrain. Growing evidence has indicated that induction of HO-1 expression via activation of Nrf2 signaling exerts neuroprotection against oxidative injury in neurons [30]. Recent studies have highlighted important roles of PGC-1*α* in neurodegenerative diseases. One such study found that overexpression of PGC-1*α* improved in motor behavior and increased in TH expression in the substantia nigra of MPTP-treated mice [31]. Our present results demonstrated that AA facilitated the clearance of MPP^+^-induced excessive ROS in astrocytes *in vitro* by reducing the expression of gp91phox and increasing the expression of the antioxidant molecules, GCLM, HO-1, and PGC-1*α*.

Which signaling pathways are involved in AA-induced enhancement of antioxidative function in MPP^+^-treated astrocytes? A previous study found that rosmarinic acid attenuated inflammatory responses through suppressing the HMGB1/TLR4/NF-kappaB signaling pathway, which may have contributed to its anti-PD-like activity [32]. Glaucocalyxin B has been shown to suppress LPS-induced PD-like symptoms via modification of TLR/NF-kappaB and Nrf2/HO-1 pathways both *in vivo* and *in vitro* [33]. In the present study, we found that AA pretreatment significantly reduced MPP^+^-induced expression of NF-*κ*B in astrocytes *in vitro*, whereas there was no significant effect on the phosphorylation levels of JNK, ERK, or p38. From these findings, we hypothesized that AA enhanced antioxidant function in MPP^+^-treated astrocytes via inhibition of NF-*κ*B expression. Hence, we subsequently inhibited the NF-*κ*B signaling pathway and explored whether the protective effects of AA remained. We found that the MPP^+^-induced expression of ROS and iNOS decreased after inhibition of NF-*κ*B, which also eliminated any such differences between the AA-pretreatment MPP^+^ group and MPP^+^ group. The antioxidant system also exhibited similar effects after NF-*κ*B inhibition in MPP^+^-treated astrocytes, in which AA pretreatment no longer restored the MPP^+^-altered expression of SOD1 and GSH. Interestingly, inhibition of NF-*κ*B did not significantly inhibit GCLM, HO-1, or PGC-1*α*. However, inhibition of NF-*κ*B eliminated the differences in these levels between the AA-pretreatment MPP^+^ group and MPP^+^ group.

*In vitro* experiments have shown that AA increases the antioxidative activity of MPP^+^-treated astrocytes. One study found that plasma rich in growth factors-Endoret (PRGF-Endoret) improved motor performance in MPTP-treated mice [34]; these effects were associated with a robust reduction in NF-*κ*B activation and nitric oxide (NO) expression in the substantia nigra. Quercetin has been demonstrated to play an important role in altering the progression of neurodegenerative diseases by protecting against oxidative stress [35]. Furthermore, dietary vitamin C intake is significantly associated with reduced PD risk [36]. However, Martinovits et al. found that systemic administration of AA for a short time (two days) does not protect mice against the dopaminergic neurotoxicity of MPTP [37]. But many researchers reported that AA exerts neuroprotective effects against MPTP-induced neurotoxic [38, 39]. Our present study demonstrated that AA alleviated pathology and behavioral deficits in MPTP-treated mice. We found that AA pretreatment increased the expression of TH in the midbrain of MPTP-treated mice. Behavioral studies—including assessments in the rotarod test, grasping strength test, pole-climbing test, and open-field test—showed that AA pretreatment ameliorated behavioral deficits in MPTP-treated mice. We found that AA pretreatment reduced oxidative stress in MPP^+^-treated primary astrocytes in vitro. Pretreatment with AA reduced MPP^+^-induced ROS and iNOS production in astrocytes in vitro, which may have been due to decreasing MPP^+^-induced expression of the NADPH oxidase subunit, gp91phox. Finally, we found that inhibition of MPP^+^-induced NF-*κ*B activation led to an increase in the expression levels of antioxidant molecules, such as SOD, GSH, GCLM, HO-1, and PGC-1*α*. Hence, our present study reveals a new role of AA in inhibiting astrocytic oxidative stress.

## 5. Conclusion

Taken together, our present study demonstrates that AA inhibits astrocytic oxidative stress in MPP^+^-treated astrocytes through suppressing NF-*κ*B, which ultimately leads to increased expression levels of antioxidant proteins to scavenge ROS to further decrease MPTP/MPP^+^-induced apoptosis. Thus, AA may represent a potential therapeutic agent for ameliorating PD symptoms. However, further research is needed to elucidate the antioxidative capacity of AA in a primate model of PD and, if efficacious, in human PD patients. Collectively, our present findings provide insight into the role of AA in ameliorating MPTP-induced PD-like behavioral deficits and pathophysiology in mice. This data suggests that AA may be advantageous in exerting neuroprotection to slow the progression of PD in humans.

## Data Availability

The data used to support the findings of this study are available from the corresponding author upon reasonable request.
